# Dietary Fiber and Processing Interventions for Enhanced Batters and Final Baked Products: A Review

**DOI:** 10.1155/ijfo/5556678

**Published:** 2026-04-27

**Authors:** Sangeeth Raj A., Praveen Kumar Dubey, Hridyesh Pandey, Ashutosh Upadhyay

**Affiliations:** ^1^ School of Agriculture, Lovely Professional University, Phagwara, 144411, Punjab, India, lpu.in; ^2^ Indian Institute of Packaging, Lucknow, 226008, Uttar Pradesh, India; ^3^ Department of Food Processing Technology, Anand Agricultural University, Anand, 388001, Gujarat, India, aau.in; ^4^ Department of Food Science and Technology, National Institute of Food Technology Entrepreneurship and Management, Kundli, 131028, India, niftem.ac.in

**Keywords:** batter, fermentation, natural dietary fiber, rheological property

## Abstract

Batter is a semiliquid mixture made up of flour, water, and other ingredients composed of biopolymers such as carbohydrates, proteins, fibers, and lipids and is used to make cake, muffins, and crepes or to coat certain foods before frying or baking, which plays a vital role in determining the texture, flavor, and overall quality of the final product. Several factors are involved in preparing batter, in which dietary fiber could be enhanced in its rheological properties, nutrition, and sensory attributes. However, fermentation improves the quality, enhances digestibility, and improves batter. Nonetheless, there is a gap in the research to fulfill this. Therefore, batter fortified with natural dietary fiber such as arabinoxylans, resistant starch, pectin, inulin, and beta‐glucan with the process interventions optimizes the rheological properties of batter and contributes to enhancing texture, stability, and overall product quality. Moreover, this review shows that soluble and insoluble dietary fiber supplementation in batter coating reduces the oil uptake during frying and decreases the fat content in various meat products. This review has studied dietary fiber limitation in batter and its interaction during fermentation and their characteristics. Also, applying dietary fiber as a nutrient‐rich source could be product‐friendly for the food industry and improve the quality and safety of batter‐based products.

## 1. Introduction

Primary causes of death worldwide are cancer, type 2 diabetes, cardiovascular diseases (CVDs), and obesity [1]. Over 3 million deaths annually occur due to improper dietary management, which leads to the chance of developing noninsulin‐dependent diabetes mellitus, along with CVDs, which are among the most severe health consequences of poor dietary habits [2]. According to the International Diabetes Federation (2021), 10.5% of the adult population (20–79 years) has diabetes, and by 2045, it is projected that approximately 783 million people will be living with diabetes. Also, the American Diabetes Association recommends that fiber intake for diabetes patients align with general population guidelines, recommending 14 g per 1000 kcal daily, or about 25 g for women and 38 g for men, with a focus on consuming at least 50% whole grains.

In response to the rising incidence of severe diseases associated with unhealthy diets and inadequate nutrition, an innovative approach has been developed to enhance food quality, consumer preferences and nutritional needs, and to address human satiety [3]. Thus, dietary fiber (DF) is widely applied in functional food ingredients and additives to develop health‐promoting value‐added products. Functional molecules, through indigestible, play a crucial role in the digestive system, aiding weight loss and promoting gut health. It enhances gastrointestinal function, supports probiotic growth, lowers blood glucose and cholesterol levels, and reduces cancer risk by decreasing fecal bile acids [4]. DF consists of SDF and IDF, which the human body cannot digest or absorb. SDF, partially soluble in water, includes substances like pectin and certain gums, while IDF, insoluble in water, comprises structural components such as cellulose, hemicellulose, and lignin [5].

DF can be classified as plant, microbial, animal, and other sources, and it may vary in physicochemical and rheological properties, as well as its SDF and TDF contents. DF is further categorized based on its solubility, viscosity, fermentation potential by gut bacteria, and origin [6]. Among these properties, viscosity, or the fiber’s gel‐forming ability, is considered a better predictor of physiological effects than solubility. However, accurately measuring viscosity presents challenges due to the variations in the fiber concentration, diet, pH, and temperature [7]. Additionally, DF is classified as fermentable or nonfermentable based on its interaction with gut bacteria, where fermentable DFs serve as substrates for colon microbiota, stimulating the growth of specific micro‐organisms and leading to the production of metabolites such as acetic acid [8].

Fermentation of batter directly influences bowel function, impacting fecal mass, stool frequency, colonic pH maintenance, and energy retrieval from nondigestible foods, depending on the fibers’ fermentation pattern. DF with poor process interventions leads to increased bulk in the large intestine, lowering the risk of constipation and colonic cancer [9]. Similarly, the physical properties of food products, such as batter and dough, play a critical role in their functionality and sensory characteristics. The drastic difference between batter and dough is based on their consistency: Dough is stiffer and moldable, while the batter is semiliquid or liquid, allowing it to flow. Dough‐based food products are generally harder to chew compared to batter‐based ones. A batter, typically a mixture of flour, water, and other ingredients, is widely used in the preparation of muffins, cakes, and crepes, as well as for coating foods before frying or baking [10]. Batters can be categorized into adhesion or interface batters, which form strong bonds between the coating and the product, and tempura‐type or leavened batters, which develop a spongy structure, making them light and easy to eat.

Various functional ingredients, including DFs, protein isolate, wheat gluten, cellulose, bran, and hulls, have been incorporated into batter formulation to enhance the quality attributes of foods [11]. Among these, DF grains such as pseudocereals, oats, and barley show greater potential in batter formulations due to their resistance to enzymatic breakdown in the stomach and small intestine, contributing to their nutritional value and supporting a healthier diet [12]. Additionally, thicker batter made from mixtures of rice, black gram, and fenugreek seeds, fermented or unfermented, is commonly used to prepare traditional foods in Southeast Asian countries. Examples include pakoda (vegetables coated in wheat flour batter), idly (steamed rice cake), paniyaram (mini idly), dhokla (fermented steamed pudding), and dosa (oval‐shaped rice and lentil crepes) [13].

Wheat flour, water, and other ingredients are commonly used in batter formulations for fried products to enhance their appearance, taste, and texture. During frying, amylose released from swollen starch granules in the batter forms a barrier, reducing water activity and limiting oil uptake into the food. To further reduce fat, cholesterol, and oil absorption, fermented functional molecules can be incorporated into meat products, thereby extending shelf life without nutritional loss. Various fibers have been supplemented in combination to formulate low‐fat meat products and emulsions. Additionally, the inclusion of functional molecules in batter coatings directly affects batter rheology by increasing viscosity, potentially decreasing cooking yield.

Rheological characterization plays a prominent role in the liquid/semiliquid food industry for evaluating DF functionality, process engineering, and quality assurance, particularly impacting batter stability and emulsions [14]. The rheological behavior of batter products is influenced by the composition of raw ingredients, spatial arrangement of components, chemical structure, microstructure, and thermomechanical properties, which boost the functionality during blending, processing, storing, frying, and baking [15]. Batter systems exhibit a shear‐thinning behavior, where an increase in the shear rate lowers viscosity, which becomes more complex with the incorporation of protein and thickeners. To address this complexity, dynamic rheology techniques are used to evaluate the relationship between the batter’s viscous and elastic components. Reference [16] performed a rheology test for cake batter incorporated with Eucheuma powder, which showed an increase in viscosity and viscoelasticity of batter with changes in the texture properties and formation of crumb color.

The market for battered and batter‐based coating products supplemented with DF‐enriched components is rapidly expanding due to consumer preferences and the desire for healthier food options [17]. Furthermore, semiliquid products are gaining substantial popularity worldwide as processed food consumption rises. According to Future Market Insights, the batter and coating market is projected to grow at a compound annual growth rate (CAGR) of 5.2% from 2022 to 2032 [18]. Numerous food industries use batters and coatings to preserve frozen foods, responding to the growing demand for convenient, high‐nutrition products. Additionally, during the appraisal period, innovative semiliquid products are prepared with clean‐label batters and proper coating, containing lower salt and fat levels. According to a report by Imarc Group, the global batter and breader premix market is projected to grow at a Compound Annual Growth Rate (CAGR) of 5.44% from 2024 to 2032 (Market Study Report, 2023) [19].

**FIGURE 1:**
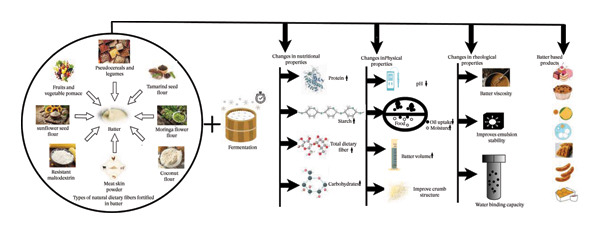
Overall fortification with dietary fibers in batter and their impact during fermentation.

Battered foods are complex systems in which the coating and the food substrate undergo significant structural changes during frying, particularly with DF and other functional components (Figure 1). In comparison, several studies focused on the addition of protein and hydrocolloids to batter formulations and their effects on rheological behavior [20]. Others have explored the formation of acrylamide during frying operations. Furthermore, researchers have developed innovative coating materials to improve the functional properties of batter‐coated products [15]. Dietary management strategies for batter formulations during frying operations have been explored by Ref. [2]. There is a notable gap in the existing literature regarding a DF limitation in batter and a comprehensive discussion on the optimization of rheological properties through DF and process interventions. Therefore, this review aimed to gather research on the limitation and effects of adding natural DF sources such as bran and hulls (fiber), inulin (DF), polysaccharides (carbohydrates), protein isolate, resistant starch (RS), resistant oligosaccharides (ROs), β‐glucan, hemicelluloses (arabinoxylans), and pectin to batter products with fermentation to optimize the rheological properties for healthier food formulations during baking, frying, steaming, and other cooking methods. Furthermore, this review addresses the effect of fermentation on batter enriched with DF to enhance the sensory attributes, physicochemical properties, textural properties, and nutritional quality. Additionally, the incorporation of natural DF has been shown to reduce oil absorption and fat content during frying operations. This review presents valuable information for food scientists and industry professionals regarding the effect of DF addition to batter‐based products.

## 2. DF and Their Classification

DFs are carbohydrate polymers that contain ten or more monomeric units that resist enzymatic breakdown in the small intestine. It is primarily derived from grains (9.76%–69.20%), vegetables (6.53%–85.19%), and fruits (16.74%–91.24%). The physicochemical properties of DF include viscosity, glucose adsorption capacity (0.17–4.65 mmol/g), cholesterol adsorption capacity (0.03–37.10 mg/g), water hydration capacity (0.95–23.90 mL/g), water adsorption capacity (2.01–25.03 g/g), and oil binding capacity (0.65–29.00 g/g) [21]. DF is further classified into SDF and IDF fibers. IDF includes cellulose, lignin, and hemicellulose and forms a network structure that physically adsorbs lead ions, reducing their toxicity and the risk of lead poisoning. SDF, including oligosaccharides and noncellulosic polysaccharides, contains carboxyl side chain groups that help reduce heavy metal toxicity [22].

DF provides numerous health benefits, such as improving gut flora, lowering blood sugar levels, and reducing the risk of obesity and CVDs. It also increases fecal volume and promotes bowel movements [21]. Studies have shown a 15%–30% reduction in cardiovascular‐related mortality, coronary heart disease, stroke, type 2 diabetes, and colon cancer among individuals with high DF consumption [23]. These benefits were observed with at least 25 g/day of DF intake, and the effect on T2D extended to higher doses (up to 45 g/day) without plateauing. In surrogate markers, DF intake significantly reduced systolic blood pressure in CVD cases and glycated hemoglobin in T2D patients [24].

Among food sources, whole‐grain cereals—especially those rich in IDF—exhibited the highest correlations with mortality from chronic illnesses. Fruits, which are high in SDF, are effective against T2D, CVD, and colon cancer; these effects remain significant even after controlling dietary habits and antioxidant intake [25]. The EAT‐Lancet Commission emphasizes plant‐based diets high in DF for both human health and environmental sustainability, as DF‐rich diets are associated with greenhouse gas emissions compared to meat production. The definition of DF has been contentious, with debates lasting two decades before the CODEX Alimentarius Commission established a formal definition in 2009 (Joint FAO/WHO Food Standards Programme). Most definitions include RS, ROs, and nonstarch polysaccharides (NSPs), which are fermented in the large intestine. Associated substances, such as lignin, phytosterols, saponins, waxes, phytates, and cutin, are often included as they contribute to DF’s functional properties [23, 26]. Regulatory agencies such as the European Food Safety Authority (EFSA), the Food and Drug Administration (FDA), and Health Canada have approved mannans, pectins, gums, β‐glucans, inulin, cellulose, hemicelluloses (arabinoxylans), RO, and RS (Canada’s Food Directorate, 2017).

DF also exhibits antioxidant properties, with polyphenols bound to DF through covalent and hydrophobic interactions [27]. These antioxidant DFs are particularly noted in cocoa nibs and husks containing hydroxycinnamic acid, gallates, dihydroxybenzoic acid, and flavonoids like quercetin and catechin [28]. The synergy between DF and polyphenols enhances DF metabolic regulatory functions, including gut barrier strengthening, glucose regulation via GLP‐1 and PYY peptides, and reduction in insulin resistance [26, 29]. DF’s role in preventing dysbiosis and supporting short‐chain fatty acid (SCFA) production further contributes to its immunomodulatory effects [30]. Despite its benefits, misconceptions about DF‐rich food persist. Many consumers associate DF primarily with processed cereals, often overlooking nutrient‐rich sources such as fruits and pulses. Under the Chilean Law on Food Labeling, 98% of ready‐to‐eat cereals were found to be high in calories, and 87% were high in sugar, despite DF‐related claims on their labels [31]. Consumer education and regulation are needed to ensure that dietary choices align with the health benefits associated with DF intake.

## 3. Fiber and Its Application to Batter‐Based Products

### 3.1. Insoluble Fibers

Insoluble fibers are derived from plant‐based sources, in which bran and hulls are primarily used in batter formulation. Bran is a by‐product of milling grains into cereals, which consists of the pericarp, seed coat, and aleurone layer. Wheat, oats, rice, and rye are the primary sources of bran, which are highly rich in fiber, minerals, vitamins, and protein content and act as a natural antioxidant, antimicrobial, and anti‐inflammatory [32]. However, the human body is not able to digest as rich in the fiber content and has a shorter shelf life. According to Tyl et al. [33], fermentation is vital in improving the digestibility and shelf life of batter products. Bran from cereals and legumes contains large amounts of cell wall polysaccharides, which are high in cellulose, arabinoxylans or pectin, arabinoxylans in wheat, and β‐Glucans in oats.

As studied by Sujarwanta et al. [34], the supplementation of 20% rice bran in meatballs improves the sensory scores with a rise in water hydration capacity, percentage of retained moisture, and retained fat. However, adding above 20% degrades the product quality. Likewise, Haque et al. [35] investigated the incorporation of 0%, 5%, and 10% rice bran in chicken nuggets, which shows no significant difference in sensory analysis and overall acceptability. Likewise, Haque et al. investigated the incorporation of 5% rice bran in chicken nuggets resulted in lower sensory scores whereas the control sample shows higher sensory score. Shanaullah et al. [36] addressed the inclusion of wheat bran with 0%, 5%, 10%, and 15% for the development of DF‐enriched chicken sausage, resulting in the finding that adding 5% wheat bran enhances the sensory analysis, flavor, and overall acceptability of the product. Meanwhile, the other three sample groups lower the lightness and yellowness values, thereby degrading the quality. Zolqadri et al. [37] investigated that the physicochemical properties of rice bran protein isolate used as a replacement for wheat flour at different levels (1% to 3%) improve the cake batter and enhance the total phenolic, amino acid content, and 2,2‐diphenyl‐1‐picrylhydrazyl (DPPH) of sponge cake.

Hulls are the outer covering of grain and seeds, which are rich in the fiber content and play a significant role in batter formulation, as discussed below. Buckwheat hull added at different amounts (10,20,30 g/hg) in steamed rice cake at cell scale (10–50 m) and tissue scale (100–500 μm) significantly affects the physicochemical and textural properties of batter [38]. Adding (20, 30 g/hg) at the cell scale results in a fluffy cake structure with decreased hardness compared to the tissue‐scale addition. Likewise, Pichler et al. [39] investigated that incorporating less than 20% oat husk positively affects gluten‐free batter properties and final bread. Also, the quality of the product is degraded, while the addition is more than 20%, which shows a negative effect on the crumb structure due to the high fiber content. Kayode et al. [40] concluded that a ratio of soybean hull to wheat flour fermented with *Aspergillus oryzae* (4%:16%) at 72 h and *Bacillus subtilis* at 72 h enhanced the nutritional quality of the biscuit without adverse effects on its physical, textural, and sensory properties. Karimi et al. [41] revealed that adding 2% pistachio green hull extract to the muffin improves the overall acceptance compared to the control. In contrast, adding 4% decreases the quality and receives the least consumer acceptance.

It was reported that the biofortification of bran and hulls effectively increases the content of essential vitamins, minerals, and other nutrients, which can be incorporated into batter products such as muffins, cake, pastries, and pies, thereby improving health benefits and providing higher nutritional value to consumers (Dash et al.). [42]. Also, Joye [43] revealed that adding DF from whole grains and bran in baking improves protein digestibility, effectively reduces starch digestibility, and lowers the glycemic value.

### 3.2. Soluble Fibers

Soluble fiber that includes β‐glucan, pectin, and inulin can be found in oats, nuts, and seeds. Research by Żbikowska et al. [44] demonstrated that replacing 20%–80% of fat with 3%–4% yeast β‐glucan in the muffin batter increases batter hardness and crumb hardness from 3.17 to 7.03 N, making crumb quality worse. Likewise, Artunduaga and Gutiérrez [45] concluded that reformulating vegetable oil with 40% beta‐glucans from *Ganoderma lucidum* reduces the fat content in cake, as it possesses fat‐mimetic properties. However, the addition of beta‐glucans generated finer crumbs and smaller cells, whereas Sharma et al. [46] reported that adding up to 5% beta‐glucan from millets to cake improves sensory, textural, physicochemical, and antioxidant properties and that quality degrades when the addition exceeds 5%. Adding more than 3% yeast β‐glucan to meat batter improves stability, emulsion capacity, and water‐holding capacity, but negatively affects sensory characteristics (Cengiz & Dogan 2021).

Pectins are widely used as a thickening, gelling agent, and stabilizer in meat products. According to Silva‐Vazquez et al. [47], incorporating 15% inulin and 15% pectin as a fat replacer in meat batter improves the emulsion stability. Likewise, Santiaguín‐Padilla et al. [48] revealed that the incorporation of 4% pectin‐encapsulated fat in meat sausage improves the hydrolytic action of lipases over triacylglycerides compared to a direct addition of pectin and without affecting its sensory acceptability. Also, crab apple peel is a rich source of pectin, which can be incorporated into sausage batter to reduce the fat content and cooking loss [49]. According to Jouki et al. [50], the supplementation of 1% pectin and 2% tomato paste into fried enriched sausage decreases the oil uptake, moisture loss, and fat content with an overall improvement in nutritional, structural, and sensory attributes when compared to the control sample. Okra pectin incorporated into beef sausage at 0.5% by Gao et al. [51] produces an acceptable sausage. Also, increasing okra pectin beyond 0.5% reduces quality, as indicated by increased hardness. According to Ahsan et al. [52], the incorporation of pectin from ripe and unripe banana peel at 2.5 and 1.5 pH into muffins resulted in pectin from unripe bananas, showing superior quality and higher acceptability in sensory evaluation due to the lower fat content. Likewise, Giarola et al. [53] studied the effect of adding sunflower meal‐pectin in egg replacement in cake batter to improve the cake crumb structure and color.

Inulin is a plant‐derived carbohydrate that can be incorporated into meat products to enhance their physicochemical, textural, and sensory characteristics. Also, it can be helpful for lipid modification and quality improvement of low‐fat meat products (Yousefi et al.) [54]. Furthermore, Montoya et al. [55] prepared pork and chicken meatballs with the inclusion of 3% inulin, which reduces the fat and shows good acceptance among consumers. Muffins prepared using 30% sugar inulin and green banana flour reduce the fat content and maintain the overall acceptability of the product, with the firmness of the cake increased, while the springiness and baking loss decreased [56]. To improve the cake’s nutritional profile, the incorporation of 2.5% oligofructose/inulin receives higher sensory scores and enhances protein, total fiber, and ash content. Also, the samples prepared with 10% oligofructose/inulin received the lowest sensory score, and quality degrades during storage [57].

Multigrain breakfast cereal batter prepared with 16% chicory root fiber (inulin) increases the DF content and decreases the fat content with improved textural properties compared to the control sample. However, the quality gets degraded when the addition is 8% and is not accepted by the consumers [58]. Incorporating 15% inulin into muffins reduces the fat and calories by 68.05% and 12.63% compared to the control, with a lower sensory score, whereas 10% inulin inclusion receives good overall acceptability among consumers [59].

### 3.3. Functional Fibers

Functional fibers are a nondigestible carbohydrate in which RS and hydrocolloids are primarily used in batter formulation and show beneficial physiological effects in humans. RS exhibits physicochemical qualities such as high water absorption capacity, lower swelling power, and higher thermal stability, which can be widely used in batter‐based products [60]. Xie et al. [61] investigated that adding RS and ice water decreases cooked pork batter’s total lipid and energy content and improves the thermal stability, gel strength, and rheological properties. The quality of the products degrades when 75% of pork fat is replaced with RS and ice water. A batter formulated with sweet potato starch for frying chicken nuggets reduces the oil uptake and moisture content, and an increase in frying time increases hardness, cohesiveness, adhesiveness, and gumminess [62]. Likewise, Matthews et al. [63] investigated that incorporating sweet potato starch and 15% protein in batter formulation for chicken frying reduced fat uptake and improved its physicochemical quality attributes. Waffle batter replaced with 12.5% banana flour and 7.5% potato starch improves the sensory, nutritional, and functional properties with a negative PRAL (−0.67), indicating that the waffle causes more alkalinity in the body when consumed [64].

Hydrocolloids are water‐loving macromolecules derived from various sources, such as microbial metabolism (xanthan gum), plant seed (guar gum), tree secretion (gum arabic), animal source (gelatin), and algae (carrageenan and alginate) with the addition of these hydrocolloids in batter products enhancing the nutritional value, sensory characteristics, and functional properties due to their application such as thickeners, emulsifier, stabilizers, and gelling agents [65]. Ghaemi et al. [66] reported that adding 0.3% XG improved the apparent viscosity of the cake batter sample and resulted in higher specific volume, porosity, springiness, and firmness than the control sample. According to Peh et al. [67], the addition of 1% methyl cellulose, 3% curdlan gum, and 1.5% guar gum into plant‐based fish cake enhanced the hardness, moisture retention, and structural and sensory properties of the cake. Hydrocolloid addition to deep‐fat‐fried meat products helped retain moisture and regulate fat absorption during frying, thereby affecting overall texture and mouthfeel [68]. To stabilize the pancake formulation, adding 5% gum arabica and 5% XG enhances the viscoelastic properties and improves the cake’s overall texture and sensory attributes [42].

## 4. Fermentation and Its Effect on Batter‐Based Products

Fermentation of batter has significant relationships with food product quality and development. Also, with the addition of DF enhances the physicochemical properties, sensory attributes, nutritional value, and textural properties. This section discusses the fermentation and its effect on batter‐based products.

### 4.1. Fermentation and Its Effect on the Physicochemical Properties of the Batter’s Product

The physicochemical properties of fermented batter are mainly composed of composite flour, which undergoes some changes in the moisture content, fiber content, batter density, batter volume, and batter stability during the processing techniques, which are discussed below.

**FIGURE 2:**
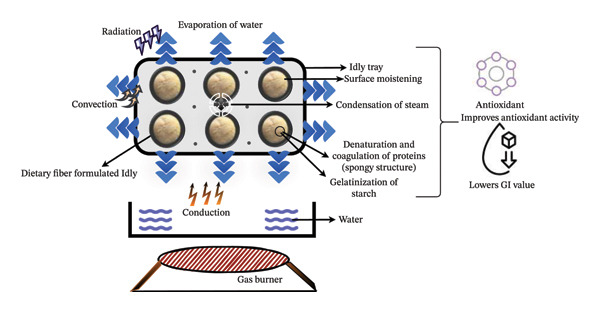
Heat transfer mechanism and solidification of idly batter during steaming. Conduction‐driven thermal energy propagation, convection‐induced steam circulation, and radiation‐surface interaction, facilitating protein denaturation, starch gelatinization, and textural aeration for an optimized spongy matrix.

Idly prepared with wheat germ through natural fermentation exhibited a significant rise in volume by 80% and a decrease in batter density from 1.02 to 0.4 g/cm^3^ as yeast and bacterial activity increased [69]. Similarly, incorporating finger millet and sorghum into fermented idly batter increases batter volume to 134% and decreases pH, resulting in the reduced bulk density and a softer texture (Figure 2) compared to the control sample (Rani et al.). [70]. Also, the addition of sorghum (35 gm), pearl millet (15 gm), finger millet (10 gm), amaranth (10%), and black gram (30 gm) to idly batter preparation significantly enhances the organoleptic score and physicochemical properties of idly compared to the control sample.

According to Bustillos et al. [71], cake density is directly correlated with batter density and then with the batter air volume fraction (∅_*a*_), and it was reported that the addition of pea protein into sponge cake batter significantly increases the cake density (*ρ* ^∗^) from (0.21 to 0.35 g cm^−3^) and reflecting less air incorporation [72]. The positive effect of fiber on food products is evident in their water absorption index, as higher fiber content increases the moisture in the crust or crumb due to its high water‐holding capacity [2]. For instance, compared to the control sample breaded fish nuggets prepared with a 1:2 ratio of XG to soybean fiber, a significantly increased moisture content was observed in the crust, core, and pickup, with moisture levels 51%, 3%, and 42% higher, respectively (Shan et al.). [73]. Similarly, battered and breaded fish balls containing 6% fermented bamboo‐shoot DF, as studied by Zeng et al. [74], exhibited increased moisture content. It can be concluded that the supplementation of fiber in fermented batter increases batter hardness and creates significant spaces for moisture movement. Dawi et al. [75] investigated that increasing the amount of locust bean pulp (carbohydrates) in cake batter improved moisture, ash, and fiber content. Similarly, incorporating 10% rye–wheat flour with fibers from beetroot, flaxseed, and buckwheat improved the bread’s physical and chemical properties [76].

For the preparation of low‐fat sausages, fat with pineapple DF and water increases emulsion stability and moisture content in all samples. Also, the protein content of the control sample was higher compared to other formulations, whereas the storage parameters, such as pH and purge, were similar to those of the control [77]. Chicken nuggets incorporated with different levels of cooked unripe plantain without peel resulted in emulsion stability, emulsion pH, moisture retention, and fat retention of meat batter increases compared to the control [78]. Also, Pereira et al. [79] observed that adding banana by‐products, which are rich in fiber content obtained from unripe bananas, increases the cooking yield in frankfurters.

### 4.2. Fermentation and Its Effect on the Nutritional Quality of the Batter’s Product

Fermented batter food products are more nutritious than their unfermented counterparts due to the action of fermenting micro‐organisms as these micro‐organisms enhance nutritional value by breaking down complex compounds and synthesizing essential vitamins and growth factors through both catabolic and anabolic processes [80]. Fermented batter products improve the protein and starch digestibility of complementary foods made from flour blends of amaranth, pumpkin, millet, and sorghum, thereby boosting their nutritional value and health benefits [81]. Also, cereals (rye, oat, barley, corn, wheat, and rice) contain specific vitamins and phytochemicals. Fermentation with LAB or yeast strains can increase the nutritional content in batter formulation. Furthermore, pseudocereals such as amaranth, quinoa, and buckwheat have greater mineral bioavailability, and incorporating them into fermented batter improves the accessibility of iron, calcium, and zinc in batter‐based products [82]. Osman and Sulieman [83] found that incorporating rice bran fiber (1.5%) and wheat fiber (1.5%) into fermented sausage increases the lactic acid bacterium (LAB) counts and decreases pH with a high water activity value during fermentation, which helps to prevent ripening and storage for a longer period during cold storage. Similarly, adding plant‐derived material (by‐products) into fermented meat products improved their nutritive and functional properties [84]. The benefits of fermentation are also evident in cereal–legume‐based products. Singh et al. [85] demonstrated that the addition of millet and black gram in a 75:25 and 70:30 ratio for a healthy millet batter resulted in increased batter volume after 16 h of fermentation and produced better sensory qualities. Incorporating fruit and vegetable by‐products into bakery products increases the batter’s water content, extends fermentation time, and produces healthier products [86]. Samtiya et al. [87] reported that fermenting sorghum flour with *Lactobacillus plantarum* increases the protein digestibility from 47% to 92%. Similar benefits were observed with quinoa flour. Fermenting quinoa flour with *L. plantarum* reduced the phytic acid content and enhanced calcium, zinc, and iron availability compared to unfermented flour samples [88]. Likewise, Sharma et al. [46] showed that fermenting Bengal gram, rice, and split beans dhokla for 18 h at 32°C increases the availability of vitamins and minerals (Ca, K, Fe) by providing a soft, spongy texture. Apart from this, fermented batter products, idly and injera, also show remarkable improvements in nutrition and texture. Idly, made from parboiled rice and black gram, fermented for 3–4 h, increases the vitamins (A, B1, B2, B12) and level of amino acids (cysteine, methionine, lysine) by enhancing the spongy structure by the denaturation and coagulation of protein in black gram, as shown in Figure 2 [89], while incorporating finger and pear millet into idly batter reduces the fermentation time from 12 to 6 and 8 h, enhancing the nutritional content, flavor, and aroma [90].

**FIGURE 3:**
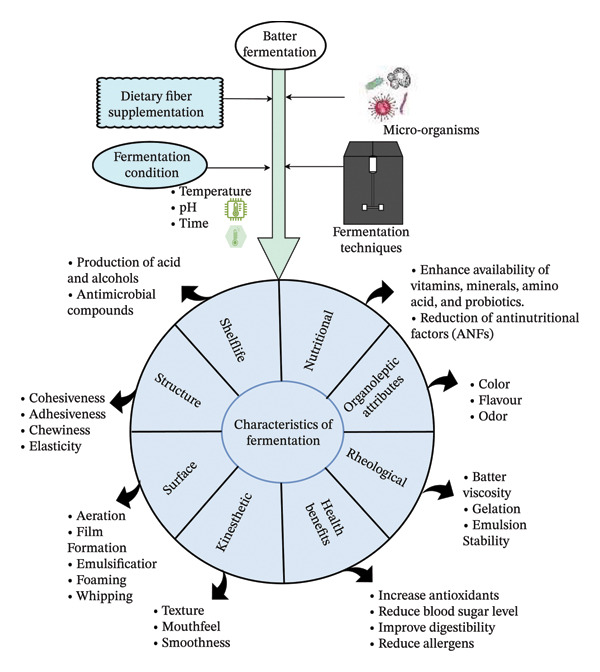
Characteristics of fermentation over batter supplemented with natural dietary fiber: Fermented better supplemented with soluble insoluble, and functional fibers, along with different LAB strain micro‐organism, enhances the final baked product by improving the rheological properties, nutritional content, and organoleptic attributes with reduction in the antinutritional factors. This mechanism paves the way for improved quality and safety of batter‐based products.

In injera, fortifying Tef batter with sorghum and carrot pulp during fermentation significantly improved iron, zinc, protein, and calcium levels while reducing antinutrients like phytic acid and tannins [91]. Also, adding *Clitoria ternatea* flower extract to fermented bread reduces glucose release and the flour’s glycemic index (GI). However, microbial fermentation is recognized for improving the functional, nutritional, and sensory attributes of batter products, offering significant potential for creating healthy, sustainable, and flavorful food items [92]. The fermentation enhances the texture, flavor, and aroma of batter products by increasing the digestibility, protein solubility, water‐holding capacity, and viscosity and making them more nutritious, as shown in Figure 3.

### 4.3. Fermentation and Its Effect on Sensory Attributes of the Batter’s Product

Sensory evaluation employs a scientific method to measure, analyze, and interpret how people respond to products through their sense of taste, touch, sight, smell, and hearing [44]. Sensory attributes of fermented batter products can be evaluated based on the nine‐point hedonic scale with the acceptability of the consumers on the sensory attributes surface feature (baking consistency, typical color, and surface cracks), crumb qualities (standard color, color uniformity, porosity), texture (firmness, elasticity, chewiness), aroma and flavor (characteristic, oily, unusual), and overall quality. Texture, an essential sensory characteristic, represents the food’s composition and reaction to applied pressure [93]. Baking transforms fluid dough or batter into a solid product, altering sensory properties and enhancing the final product’s quality, taste, aroma, and texture [94]. For instance, the savory version of fermented paniyaram batter made of rice, black gram, and foxtail millet had higher overall acceptability compared to the sweet version [95].

The development of muffin batter with soybean protein replacing egg and milk affects the texture of baked goods, making them softer and springier than the control. Likewise, the same result was obtained when pea protein was added to sponge cake batter by Bustillos et al. [71], which improved the sensory property and its structure during baking. The addition of egg white solids into bread batter decreases cohesiveness and shows a better acceptability of sensory attributes [96]. The bread prepared with wheat flour supplemented with 15% mushroom powder obtained the lowest sensory score (7.14), whereas bread made with 100% wheat flour showed a higher score (11.71) [97]. The same result was obtained by Ibrahim et al. [98], while incorporating 15% mushroom powder into the cake batter significantly improves sensory attributes such as color, flavor, and texture, which was equally acceptable to the control cake.

Batter formulation enriched with alternative flours exhibits diverse sensory outcomes. Cake made with 5% baobab pulp flour showed improved taste, but increasing substitution levels to 10% affected the overall sensory quality [99]. Incorporating 20% Bombay locust powder into cake batter produced a protein‐enriched product with good sensory scores [100]. Similarly, muffins fortified with grasshopper powder increase nutritional value and acceptable sensory attributes, with softer crumbs and good overall acceptability scores [101]. Sensory attributes of bakery products showed better results while incorporating 5% brewer’s spent grain (BSG) on different flours [102]. Supplementation of wheat flour, soybean flour, and pomegranate flour into the cake at different ratios 85:10:5, 82.5:10:7.5, and 80:10:10 by Ayoubi et al. [103] resulted in an overall acceptability mean score of 7.80, 7.74, and 7.96, serving as a functional food with good health potential.

Incorporation of ground and soaked pigmented rice (black/red) as a replacement for white rice in idly batter changes the nutritional properties and organoleptic characteristics of idly, such as adhesiveness, cohesiveness, and resilience, which had lower values (Susmitha et al.) [104]. Fermented idly batter made with different dietary levels of pearl millet flour, such as 20%, 40%, 60%, 80%, and 100%, shows the overall acceptability scores as 7.1, 7.0, 6.4, 6.1, and 6.0, where the 100% PMF received the lowest score, indicating that it is not commercially viable as a product [105]. Adding curry leaves to idly during a 12‐h fermentation period led to improved sensory scores over control idly, with these attributes preserved even after 5 days of batter storage [90].

Dosa batter prepared with 30% rice + 40% little millet + 20% black gram + 10% lentil showed an overall acceptability score of 8.5, making the millet dosa mix batter more nutritious and with good sensory properties [106]. The development of a low‐fat muffin with 50% avocado puree as a fat replacer was acceptable across all sensory attributes, whereas adding more than 50% negatively affected organoleptic characteristics, resulting in lower scores [107].

Substituting 10% of wheat flour with button mushroom powder in sponge cake batter resulted in good sensory attributes, whereas the control, 5%, and 10% were in the range of 5.8–7.3, indicating moderately acceptable [108]. Muffin batter formulation containing a 1:1 legume‐waxy rice mixture resulted in a sensory score of 6.0, which is liked slightly by consumers, and it could be a promising alternative to wheat flour for making gluten‐free bakery products [109]. Meanwhile, muffins prepared at 50:50 and 65:35 showed no statistical difference in overall acceptability compared to control muffins. Also, the incorporation of 40% β‐glucan in the muffin resulted in a decline in product quality both during baking and after storage [44], and the addition of 60% or more resulted in increased batter hardness and decreased crumb quality during baking and storage. According to Salazar et al. [110], the incorporation of banana flours into sausages results in higher hardness and a darker color and also preserves microbial growth (bacteria, molds, and yeast) with acceptable sensory properties; this growing acceptability over time reflects rising awareness of sausages. Likewise, adding 25% coconut flour to the batter to prepare muffins improves the sensory attributes, physicochemical characteristics, and nutritional value [111].

### 4.4. Fermentation and Its Effect on the Textural Properties of the Batter’s Product

The textural properties of fermented batter products, such as idly, dosa, dhokla, uttapam, jalebi, and cakes, were studied using texture profile analysis (TPA). This can be either linear (for springiness) or sigmoid (for hardness). This hardness can also be associated with an increase in the amount of granular lipoprotein in the batter. It is affected by the degree of aeration of baked products [112]. The texture plays a crucial role in determining the quality of fermented batter products, as it is influenced by microscopic and macroscopic properties [113].

Gluten‐free muffins incorporating rice sourdough fermented with *L. spicheri* DSM 15429 resulted in rapid growth of micro‐organisms in batter formulations, which influenced the textural properties, volatile profile, and sensory characteristics of the muffins, with firmness and smoothness softening. At the same time, fluffiness and bounce improved their parameters (Chiş et al.). [114]. Likewise, Nami et al. [115] described that pearl millet sourdough fermented with LABs can improve baked products’ mouthfeel and organoleptic properties. Also, Gobbetti et al. [116] reported that LAB plays a vital role in enhancing the consistency, and organoleptic and nutritional composition of final products. Micro‐organisms such as starters, bacteria, yeast, and mold cultures play a crucial role in meat fermentation, enhancing texture change, flavor formation, and biogenic amines accumulation [117].

Using *S. xylosus* in fermented meat products, which can act as a good starter culture, improves the texture, taste, color, odor, and overall acceptability of meat products [118]. Fermentation in meat products improves organoleptic characteristics and also enhances the mouthfeel and taste of cured and partially fermented sausages [119]. Likewise, the growth of LABs in fermented sausages enriched with varying levels (0%, 1%, 2.5%, and 5.0%) of processed caustic orange albedo (DF) resulted in increased hardness, springiness, gumminess, and chewiness values [120].

## 5. Fermentation Characteristics in Fiber‐Fortified Batter Products

Generally, wheat flour, composed of starch and protein, is used to prepare batter or dough products. The addition of DF significantly impacts the fermentation characteristics by disrupting the protein–starch network, which hinders gel structure formation and modifies rheological properties [121]. The fermentation process itself improves digestion and nutrient absorption by reducing the availability of simple sugars and carbohydrates [122]. Yeast and LABs enhance fermentation efficiency and product quality; when fermented batter is fortified with DF, it further supports microbial growth and enzyme activity, thereby boosting metabolic performance [28]. This process naturally enriches the batter with vital macromolecules such as vitamins, proteins, and essential amino acids, while enhancing organoleptic properties including color, flavor, and aroma. Furthermore, fermentation contributes to food safety and reduces the energy required for cooking [46].

**FIGURE 4:**
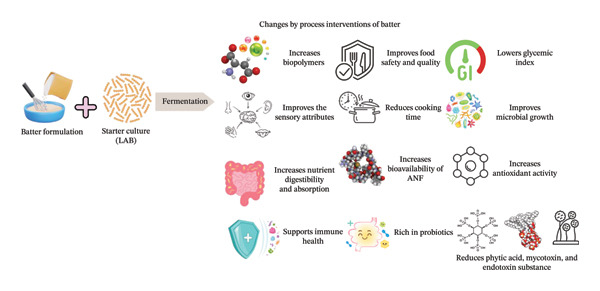
Fermented batter formulations and unlocking its health benefits: Impact of fermentation on batter with lactic acid bacteria as a starter culture transforms batter into a more functional and health‐beneficial food product.

According to Nkhata et al. [123], the composition of substrates, the specific fermenting micro‐organisms, pretreatment methods, and duration are the defining factors of the fermentation process. In fiber‐fortified batter and meat‐based products, LABs typically dominate, producing beneficial enzymes and preventing the growth of harmful pathogens [124]. Specific characteristics and outcomes of fermentation (Figure 4) in fiber‐rich batter systems include an extended shelf life for products like bakery goods and patties and enhanced organoleptic profiles where fiber incorporation improves texture and sensory appeal. Additionally, fermentation deactivates unwanted substances in plant‐based fibers and enhances nutritional values through rapid microbial growth, creating unique profiles resulting from enzymatic activity. This process also increases the in vitro antioxidant capacity and significantly shortens cooking time.

Fermentation synthesizes vital antioxidants, increases vitamin bioavailability, and generates bioactive peptides and enzymes. Specifically, LAB strains such as *L. paracasei*, *Lb. rhamnosus*, and *S. cerevisiae* have been shown to increase the total phenolic content in grains such as quinoa, buckwheat, and barley [125]. In bakery goods, sourdough fermentation is essential for acidification and proteolysis, producing aromatic compounds and conferring antihypertensive properties [126]. These natural antioxidants produced during the process are increasingly preferred over synthetic alternatives due to safety considerations [127].

The biochemical characteristics of the process are marked by the release of glucose, which serves as a favored substrate for micro‐organisms, typically leading to a decrease in the total carbohydrate content after 24 h [128]. In certain grain varieties, fermentation decreases starch content while increasing carbon dioxide and ethanol production. However, prolonged soaking in preparations such as idly batter may reduce phytochemical levels due to leaching [123]. Ultimately, the production of lactic and acetic acids by LABs reduces the batter’s pH below 4.0, effectively inhibiting harmful micro‐organisms and creating a final product with superior nutritional and sensory properties compared to the raw ingredients [129, 130].

Lactic fermentation defines the core characteristics of batter products like idly and dosa by utilizing LABs to enhance nutritional density and sensory appeal [129]. This process is characterized by the systematic removal of undesirable mycotoxins and antinutrients while enriching the matrix with bioactive metabolites such as probiotics, antioxidants, and phenolics. These biochemical shifts significantly improve food safety and develop the signature tangy aroma and refined texture found in diverse staples across India and Africa. Ultimately, these fermentation characteristics transform raw ingredients into functional foods with superior shelf stability and health‐promoting properties [131].

Generally, meat batter fermentation can be classified into controlled and uncontrolled processes, in which a starter culture is introduced to produce the desired outcome. In terms of sensory characteristics, uncontrolled fermentation is superior to controlled fermentation. Reported that the addition of DF includes animal fats, employing probiotic micro‐organisms for fermentation and prebiotic fiber to traditional formulations to enhance functional properties and promote healthier meat [132]. Fermentation reduces pH and produces bacteriocins and bioactive peptides to prevent the growth of pathogenic and spoilage micro‐organisms, contributing to improved food safety in meat products [133].

In baking, fermented proso millet bran (DF) improves antioxidant and nutritional qualities while reducing the GI of cake batter, as illustrated in Table 1 [134]. Wang et al. [38] revealed that the fermentation of cereals simultaneously improves flavor, aroma, texture, nutritional quality, and extends the shelf life of baked goods. Yeşil and Levent [135] concluded that the preparation of gluten‐free bread formulation using yeast in fermentation gives a soft crumb texture and enhances symmetry, pore structure, flavor, and aroma, as well as overall acceptability compared to spontaneous fermentation. However, gluten‐free bread prepared through sourdough fermentation improves sensory, nutritional, and textural attributes [150]. Ding et al. [136] demonstrated that rice starch in bread batter fermented at 35°C for 38 min improves the baking and texture attributes, color, and crumb grain characteristics. The addition of different proteins in fermented rice cakes exhibits strong elastic and weak viscous gel behaviors. These reduce hardness and crumb texture firming rate, increase springiness, enhance organoleptic parameters, and retard amylopectin recrystallization [137].

**TABLE 1:** Fermentation and natural dietary fiber effects on the batter‐based products.

Foods products	Dietary fiber source	Fermentation condition	Interaction between fermented dietary fiber and batter	Outcome of fermentation	References
Cake	Proso millet bran	—	✓ Reduces elasticity of the batter and increased hardness. ✓ Lowers viscosity and cohesion with acceptable texture.	✓ Increased phenol content and DDPH. ✓ Lower glycemic index with maintaining sensory qualities.	Xiao et al. [134]

Bread	Buckwheat, quinoa, amaranth	30°C for 20 h.	✓ Increases water absorption and changed dough consistency. ✓ Higher ash and protein content with more fermentation	✓ Improved phenolic, antioxidant, and mineral content. ✓ Yeast: softer crumb ✓ Spontaneous: Harder, denser texture.	Yeşil and Levent [135]

Bread	Rice flour and corn starch	38 min at 35°C and 85% humidity	✓ Produces homogeneous dough, impacting the bread’s texture and volume ✓ Good expansion characteristics.	✓ Hardness decreased with increase in the loaf volume and porosity. ✓ A significant decrease in springiness and chewiness.	Ding et al. [136]

Rice cakes (RCs)	Soy protein isolate (SPI), whey protein concentrate (WPC), marine fish collagen (MFC)	30°C for 5 h and 30°C for 3 h	✓ Changes viscoelastic properties, ✓ Increases porosity, specific volume, and reduced crumb hardness.	✓ Improved sensory quality, and extended shelf life, especially with MFC.	Meng & Kim [137]

Biscuit	Finger millet	28°C for 24, 48, 72 h	✓ Modified the chemical composition, and influences moisture and texture properties ✓ Boost Maillard reactions.	✓ Increased protein, carbohydrates, energy values, mineral content, and antioxidant activity. ✓ Enhanced sensory properties.	Mudau et al. [138]

Muffin	Quinoa flour, inulin, buckwheat flour	30°C for 24 h	✓ Influences carbohydrate metabolism, increased lactic acid, and acetic acid content, ✓ Altered texture and viscosity of the batter.	✓ Enhanced folic acid content and mineral bioavailability. ✓ Improved sensory properties and shelf life of muffins.	Chiş et al. [114]

Bread	Teff, rice, corn, and sorghum	37°C for 24 h	✓ Improved dough elasticity, reduced dough softening, and enhanced water absorption.	✓ Increased specific volume, and improved texture ✓ Improve the structure of gluten‐free bread.	Chochkov et al. [139]

Muffin	Rice sourdough	30°C for 24 h	✓ Improve nutritional profile through spontaneous fermentation.	✓ Improve textural and sensorial properties of gluten‐free baked muffins.	Chiş et al. [140]

Muffin	Green banana flour	—	✓ Influences the dough’s water retention, resulting in a moist texture and a stable structure postbaking.	✓ High protein content, moderate antioxidant activity, good sensory acceptability (84.5%), and high protein digestibility.	Radünz et al. [141]

Bread and muffin (partially gluten)	Hairless canary seed, wheat, corn	—	✓ Phenolic acids in bound form reduced, unbound phenolic acids increased. ✓ Improved antioxidant activities in both bread and muffins	✓ The DPPH, ABTS, and ORAC antioxidant activities improved in breads and muffins	Abdel‐Aal and Rabalski [142]

Muffin	Grape pomace powder (GPP)	—	✓ Decreases dough volume and increased strength, reducing gas cell retention. ✓ Enhances nutritional value with higher fiber and polyphenol contents.	✓ Increased fiber, lipid, and nutritional content with improved sensory properties.	Baldan et al. [143]

Muffins	Almond flour and oat fiber	NA	✓ Impacted water activity and crust color. ✓ Acceptable sweetness levels achieved with stevia.	✓ Overall rating scores of the control muffins were higher than those containing rare sugars (*p* < 0.05)	Moss et al. [144]

Cake (gluten‐rich)	Wheat flour	37°C for 0, 3, 6, and 9 h	✓ Less air retention in the batter.	✓ Improved flavor and crust color; decreased volume, height, specific volume, and baking loss; more compact texture with higher hardness, gumminess, and chewiness.	Jia et al. [145]

Bread	Chia and flaxseed sourdoughs	30°C for 24 h	✓ Altered the dough structure by increasing water absorption and improving the dough’s rheological properties. ✓ Improved volume and visual appearance of breads	✓ Improved specific volume and sensory characteristics.	Maidana et al. [146]

Rice cakes (RCs)	Sourdough with Dry Yeast (SDDY) and Sourdough with Makgeolli (SDMG)	30°C for SDDY: 3 h, 30°C for SDMG: 5 h	✓ Increased moisture content and acidity, affecting dough structure and firmness	✓ Improved texture, extended shelf life by reducing hardness and staling rate, better sensory qualities.	Meng et al. [147]

Cake	Wheatgrass powder and ∝‐amylase	37°C for 4–7 h	✓ Minimal effect on fermentation. ✓ α‐Amylase addition of 0.03, 0.05, and 0.07 wt% increased the rate of fermentation and shortened the fermentation period.	✓ Improves antioxidant properties of fortified cakes. ✓ Good sensory acceptability for 0.05	Pham and Le [148]

Sponge cake	Pulse flours (lentil, chickpea, lupin, green and yellow pea)	NA	✓ Affected lipid oxidation and Maillard reaction	✓ Attractive nutritional and structural properties.	Krause et al. [149]

The effect of spontaneous fermentation on finger millets for 24 h at 30°C in cookies resulted in increases in nutritional content and antioxidant activity (DPPH and FRAP) [138]. Similarly, spontaneous fermentation has been shown to enhance the protein content and DF in pearl millet bread and foxtail flour [115]. Longoria et al. [151] observed that increasing fermentation period in cassava dough with Lactobacillus paracasei for biscuit preparation increased protein content and decreased fat content. The fermentation of quinoa flour with *L. plantarum* ATCC 8014 for 24 h enhances the macromolecules and rheological qualities of the muffin, as glucose, maltose, and fructose were metabolized, resulting in an increased lactic acid content [140]. Adding *L. spicheri* DSM 15429 on a gluten‐free muffin with rice sourdough fermentation for 24 h at 30°C (Table 1) by Chis et al. [114] resulted in micro‐organisms able to grow in rice flour and influence the texture and volatile profile of muffins, as well as their sensory characteristics, where the hardness and cohesiveness decreased, while resilience and springiness improved. Strains like *Enterococcus durans* and *Pediococcus pentosaceus* positively affect the baking characteristics of batter or dough products [139].

Similarly, muffins prepared with green banana flour have good sensory acceptability, high protein digestibility, and considerable antioxidant potential compared to other flours [141]. Abdel‐Aal and Rabalski [142] showed that muffins and bread made from hairless canary seeds (amino acids) enhance the baking procedure and antioxidant assays such as DPPH, ORAC, and ABTS. Mixing and fermentation of dough products induce more significant changes in phenolic acids than baking, primarily due to enzymatic and microbial activities [152]. Incorporating (15% and 25%) grape pomace powder in fermented batter improved the nutritional composition and fiber content in the muffin [143]. Also, incorporating agavin‐type fructans (ATFs) in fermented batter reduces the sucrose and fat contents [153]. Likewise, to produce low‐carbohydrate muffins, the substitution of flour and sucrose with almond flour, whey protein concentrate, oat fiber, resistant maltodextrin (RM), and d‐allulose or d‐tagatose resulted in improvement in nutritional profile and sensory attributes [144].

**FIGURE 5:**
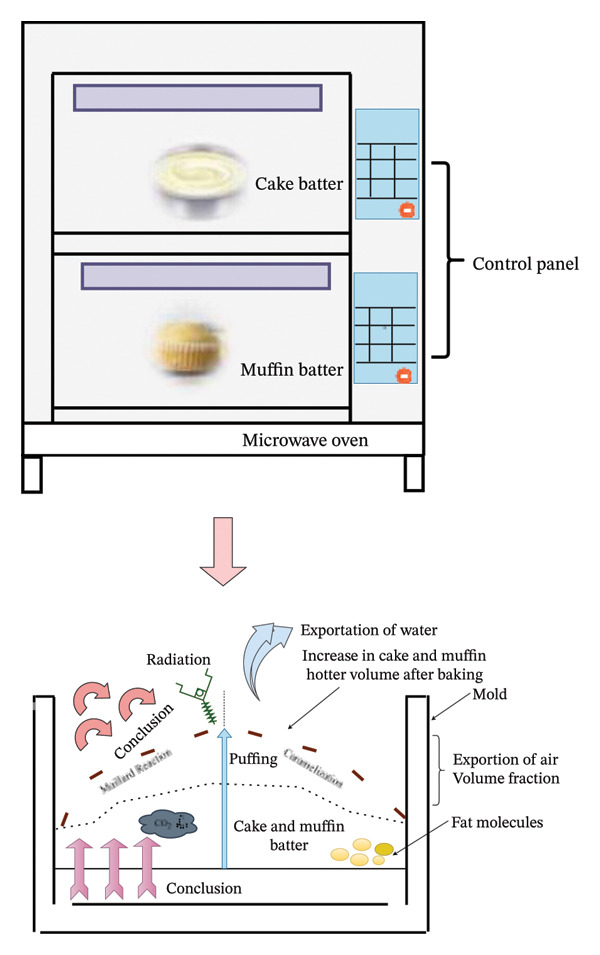
Heat transfer and chemical reaction in the cake‐ and muffin‐baking process: The application of heat into batter causes a moisture reduction and structural expansion with distinct outer layer. Also, the chemical reaction of biological macromolecules present in batter contributes to color formation, texture enhancement, and gas release, leading to an overall increase in volume and improved sensory attributes.

Fermenting egg white for up to 9 h positively influenced the cake quality, enhancing flavor and crust color while reducing baking loss [145]. However, Lee et al. [154] revealed that fermenting egg white increased the specific gravity in cake batter due to reduced air. Incorporation of sourdough fermented chia and flaxseed in gluten‐free breads increases proteins, DF, and essential fatty acids, such as ω‐3 linolenic acid content, while reducing the intake of saturated fatty acids [146]. Rice cake fermented with yeast or probiotic makgeolli improves the sensory properties but does not significantly affect appearance, flavor, and taste [147]. Cake made with fermented pulse flour displays sponginess, with desirable nutritional and structural properties, as shown in (Figure 5). Carbohydrate fiber, starch, and protein content, while baking, activated the Maillard reaction and caramelization, leading to an increase in pyrazines and furanic compounds in the pulse‐based products [149].

## 6. Fortification of Fermented Batter Products With Fiber

Recently, DF from different sources has been partially or totally replaced to increase the total DF content in food products. Supplementation of buckwheat as a DF source for fermented batter products acts as a functional ingredient in batter formulations, as they are rich in nutritional values, as shown in Table 2 [169]. Buckwheat has a well‐balanced amino acid composition and is also rich in antioxidants, such as isovitexin, vitexin, orientin, rutin, and quercetin, which exhibit anti‐inflammatory, antihypertensive, antidiabetic, antibacterial, and anticancer effects. Fermentation reduces antinutrient components, such as phytic acid, and enhances digestibility by decreasing nondigestible carbohydrates [170]. Fruit and vegetable waste can provide DF that, when incorporated into food formulations, contributes a high concentration of proteins, vitamins, minerals, polyphenols, and fiber, making the products more nutritionally beneficial [171]. Generally, DF is from the edible part of plants and agricultural products. Apart from that, meat and chicken skin can also be utilized to reduce the fat content in food products as chicken skin contains 3% collagen, which has the property of water binding capacity and modifying texture ability due to collagen characteristics. Thus, the chicken skin can be supplemented as a DF source, as a fat replacer [165].

**TABLE 2:** Batter‐based product fortified with natural dietary fiber and their factors.

Batter products	Intrinsic factors constant	Dietary fiber source	Actual fiber	Total dietary fiber level	Frying condition (°C/time)	Moisture content	Oil reduction rate	References
Goat meat nuggets	Ingredients (lean meat, common salt, sodium nitrite, sucrose, sodium tripolyphosphate, ice flakes, whole egg liquid, refined vegetable oil, condiment mix, and spice mix)	Amaranth and quinoa flour	—	1.5% and 3%	80 ± 2℃ for 35 min	66.22 ± 0.17 65.63 ± 0.15	13.15 ± 0.11 13.53 ± 0.14	Verma et al. [155]

Muffin	Ingredients (wheat flour, sugar, sodium bicarbonate, and ammonium hydrogen carbonate, oil, egg, and milk)	β‐Glucan	Soluble	3% and 4%	175°C for 17 min	21.20 ± 1.32 24.04 ± 1.45	60% and 80% fat reduction	Żbikowska et al. [44]

Cake	Ingredients (rice flour, egg, sugar, sunflower oil, milk, and baking powder)	APP (apple pomace powder) OPP (orange pomace powder) CPP (carrot pomace powder)	Soluble and insoluble	5% 10% 15%	170°C for 40 min	APP 16.71 ± 0.86 16.23 ± 2.49 17.60 ± 1.38 OPP 18.36 ± 0.58 17.80 ± 0.99 16.88 ± 1.42 CPP 15.54 ± 0.29 16.78 ± 0.37 16.51 ± 1.25	APP 22.96 ± 0.19 23.31 ± 0.65 23.65 ± 0.08 OPP 23.48 ± 0.37 23.76 ± 0.20 23.17 ± 0.14 CPP 24.25 ± 0.33 23.53 ± 0.12 23.10 ± 0.07	Kırbaş et al. [156]

Chicken nuggets	Ingredients (chicken breast, wheat flour, corn flour, rice flour, salt, leavening agent, and water)	Defatted rice bran	Insoluble and soluble	15%	185°C for 6 min	50.74 ± 2.69	6.12 ± 1.17	Chayawat and Rumpagaporn [157]

Muffin	Ingredients (whole wheat flour, baking powder, salt, egg, milk, vegetable oil, and granulated sugar)	Brewers spent grain (BSG) extracted from barley grain	Soluble and insoluble	15%	176.7°C for 10 min	1.33 ± 0.04	—	Shih et al. [158]

Chicken nuggets	Ingredients (refined wheat flour, sodium nitrite, poly‐phosphate, dry spice mix, and condiments)	Moringa flower	Soluble and insoluble	1% and 2%	100°C for 40 min	66.36 ± 0.82 65.74 ± 0.56	—	Madane et al. [159]

Chicken meat balls	Ingredients (lean meat, vegetable oil, salt, spice mix, condiments, and water)	Peral millet flour + wheat flour grape pomace powder + pomegranate pomace powder Carrot pomace powder + beetroot pomace powder	Insoluble and soluble	3.50% + 5.00% 2.00% + 1.00% 1.00% + 1.50%	80°C for 20 min	66.02 ± 0.85 68.14 ± 0.11 66.33 ± 0.23	—	Santhi et al. [160]

Pork meat balls	Ingredients (pork lean, pork fat, egg, ice water, salt, sugar, and white pepper)	Kiwi fruit pomace	Insoluble	3%	—	63.77 ± 1.39	17.87 ± 1.08	Zhao et al. [161]

Waffles	Ingredients (flour, water, salt, sodium bicarbonate, ammonium bicarbonate, palm oil, and lecithin)	Wheat + rye, oat, barley, corn	Soluble pentoses, arabinoxylans, and β‐glucan	80% + 20%	170°C for 2 min	13.30 ± 0.03 13.82 ± 0.41 13.95 ± 0.64 12.20 ± 0.16 12.00 ± 0.14	—	Nasabi et al. [162]

Peanut batter–coated nuts	Ingredients (wheat flour, pregelatinized corn starch, resistant starch, salt, and baking soda)	Sunflower seeds	Insoluble‐resistant starch	10%	170°C for 120 s	SC = 3.03% SPI = 3.30%	54.24% 61.30%	Wang et al. [163]

Waffles	Ingredients (chickpea flour, spent grain flour, wild garlic paste, golden flax seed, hemp seeds, leaving agents, and salt)	Brewers spent grain	Soluble and insoluble	Control sample Spent grain—10%	—	5.86 ± 0.02 5.88 ± 0.02	—	Chetrariu & Dabija [164]

Chicken sausage	Ingredients (chicken breast, ice water, pork‐back fat, sodium chloride, sodium tripolyphosphate, sodium nitrite, isolate soy protein, monosodium glutamate, and spice mix)	Chicken skin and wheat fiber mixture	—	5% 15%	—	64.87% 68.9%	Fat Content 16.57% 16.72%	Choe and Kim [165]

Frankfurters	Ingredients (pork meat, pork‐back fat, water, sodium chloride, sodium tripolyphosphate, and sodium nitrite)	Sunflower seeds	Soluble‐nonstarch polysaccharides and insoluble	2% 4%	Smokehouse	64.99 ± 0.19 64.47 ± 0.07	—	Grasso et al. [166]

Muffin	Ingredients (wheat flour, honey, butter, baking soda, and egg)	Coconut flour	—	0% 5% 10% 15%	180°C for 25–30 min	20% 19.80% 19.30% 18.70%	—	Ramya and Anitha [111]

Cake	Ingredients (wheat flour, rice flour, milk, egg, sunflower oil, sugar, and baking powder)	Tamarind seed gum	Soluble	0.4%	180°C for 35 min	26.61 ± 0.68	12.22 ± 0.51	Monthe et al. [167] and Wu et al. [168]

Frankfurters	Ingredients (beef, pork, pork‐back fat, ice, wheat flour, sodium chloride, sodium nitrite, polyphosphates, and ascorbic acid powder)	Green banana peel flour	Soluble‐ pectin and inulin Insoluble‐resistant starch	8%	73° for 20 min	55.12 ± 0.07	12.69 ± 0.01	Salazar et al. [110]

Fish balls	Ingredients (wheat flour, glutinous rice flour, egg white powder, baking powder, salt, deionized water, and breadcrumbs)	Bamboo shoot	Insoluble	6%	170°C for 50 s	36.12 ± 0.13	17.65 ± 0.40	Zeng et al. [74]

Naturally available seaweed DF SDF), which is extracted from red algae, plays a vital role in biological activities and exhibits beneficial effects such as antimicrobial, antioxidant, and antiviral activity, which can perform as an emulsifier stabilizer in meat products, where prebiotics give outstanding properties like water‐holding capacity and swelling by decreasing the cooking loss and increasing final yields [172]. Supplementation of sunflower seed with RS helps to improve the oil uptake of batter‐coated products, which helps to make the product denser and more uniform in shape [163].

Verma et al. [155] investigated adding 1.5% amaranth and 3% quinoa seed flours in batter formulations for frying goat meat nuggets, which impacts batter stability by reducing the moisture content to 65%–66% with an increase in the fat content and shows a higher elastic modulus and lower storage modulus compared to the control. Muffin hardness is inversely related to volume. Incorporation of 5%, 10%, and 15% pomace powders (apple, orange, and carrot) increases specific gravity, fiber, protein, ash, and sugar contents in cake batter. 10% carrot pomace powder yielded the highest apparent viscosity, while increasing fiber sources decreased cake volume. 5% orange pomace powder, with its high total DF, was noted as a valuable DF source [156].

Incorporating 15% crude protein of defatted rice bran (DRB) into chicken nugget batter increases total DF (1.24%–3.06%) and moisture while keeping the inner batter moist and reducing oil content, creating a crispy crust layer of nuggets, whereas 20% addition resulted in a lower score on overall acceptability [157]. Likewise, the same result was found by Madane et al. [159]. When 2% *Moringa oleifera* (protein and DF) was incorporated in chicken meat nuggets, it improved the odor scores and oxidative stability by reducing lipid oxidation during storage, whereas 1% addition did not show any changes in the product identical to the control sample. Also, Santhi et al. [160] performed chicken meatballs with grape pomace (2%) + pomegranate pomace (1%), and carrot pomace (1%) + beetroot pomace (1.5%), which reduced moisture content compared to the control, while pearl millet (7%) + wheat flour (10%) produced the highest yield and grape pomace and pomegranate pomace‐maintained yields similar to the control. Zhao et al. [161] found that adding 3% insoluble DF from kiwi fruit pomace into low‐fat pork meatballs increased the moisture content, where 7% increases hardness, while control samples had the highest flavor and texture scores. Adding 15% BSG to muffins decreases the moisture content by 1.33% and increases protein (13%), TDF (23%), SDF (9%), and IDF (12%). BSG 20 muffins look darker in color with affected flavor and texture, which was less acceptable by consumers. However, adding BSG 10 does not affect the product [158]. Ramya and Anitha [111] included 25% coconut flour and honey into muffins, which enhances the nutritional value, sensory, and physicochemical characteristics by reducing the moisture content with improved texture, taste, aroma, and overall acceptability and observed that while the addition is between 5% and 15%, quality degrades with a low acceptable taste.

According to Nasabi et al. [162], incorporating oat, barley, rye, and corn flour in wafer sheets increased essential amino acid and phytochemical contents, with rye having low gluten and high DF and producing the darkest wafer. Corn shows more yellowness due to carotenoid; likewise, oat and barley show redness due to the Maillard reaction. Adding 20% rye and barley increased moisture contents (2.6% to 4.13%) compared to the control. Adding RS and 6% soy protein isolate (SPI) to the batter of fried nuts reduces oil uptake and improves overall quality. SPI resulted in a higher moisture content, decreasing the oil content in peanuts and sunflower seeds by 17.98% and 15.69%, making the batter more homogeneous and denser [163]. According to Chetrariu and Dabija [164], incorporating spent grain (amino acids) into wafer batter formulations shows a darker wafer color and a heterogeneous pore distribution with large pores in the center. The incorporation resulted in increased ash, protein (11.17%), and dietary fiber (11.64%) contents, with no significant changes in moisture levels comparable to the control. The inclusion of sunflower seed flour at 2% and 4% levels into frankfurters increases lipid interactions and protein and mineral contents, decreases the fat content, and makes the product darker, enhancing phenolic compounds. The 4% sunflower seed flour exhibited the highest lipid interaction, resulting in greater lipid disorder than the control [166]. However, Choe and Kim [165] used the combination of 5% and 15% chicken skin and wheat fiber mixture (CSFM) as a fat replacer in chicken sausages, resulting in higher moisture, lower fat content, and improved quality characteristics.

## 7. Effect of Rheological Properties of Batter Enriched With DF Formulated Product

Incorporation of antioxidant‐rich DF (ADF) with process interventions into food formulations, which consist of biopolymers, significantly influences the rheological properties of flow behavior and viscoelasticity of batter, which in turn impacts the quality attributes of batter‐based products such as crumb, crust formation, texture, and volume. Further properties such as moisture retention, ingredient blending, and batter expansion impact the texture and baking behavior on the basis of DF sources and fermentation period, as shown in Table 3 [179]. Characterizing healthier batter formulations is crucial for ensuring technological quality, involving textural, rheological, thermal, structural, and microscopic analyses. These parameters directly influence the final product’s texture, sensory characteristics, and consumer acceptability [180]. Viscoelastic properties of the batter’s viscosity enable it to retain air bubbles during processing. The air volume fraction, or porosity (Φ_*a*_), is strongly linked to the batter’s rheological behavior, impacting the final product’s quality. Also, rheological properties describe how the batter responds to applied stress through flow or deformation, which is essential due to its complex structure [71]. Rheological properties can be classified into empirical (descriptive) and fundamental (basic) methods, both of which are used to monitor the batter’s response to applied stress during processing. However, empirical techniques are strong for wheat doughs. They are still being developed for gluten‐free batters [181].

**TABLE 3:** Antioxidant‐rich dietary fiber effects on physical property of fortified batter‐based products.

Antioxidant‐rich dietary fiber	Food products	Effect on batter	Effect of viscosity	Effect of hardness	Antioxidant capacity	References
Eucheuma (20%)	Cake	✓ Reduces the release of metabolites such as amino and fatty acids. ✓ Steady shear flow showed the consistency index decreased.	Decreased	—	Increased	Huang and Yang [16]

Drumstick (1.0% and 2.0%)	Chicken nuggets	✓ Improves the oxidative stability and odor scores. ✓ Better gumminess and chewiness compare to control.	—	Decreased	Increased	Madane et al. [159]

Dragon fruit peel (1.5% and 3%)	Chicken nuggets	✓ Improves the emulsion stability and cooking yield. ✓ Decreases lipid oxidation and improve odor scores.	—	Decreased	Increased	Santhi et al. [160]

Mushroom powder (10% and 15%)	Cake and sausages	✓ Better organoleptic aspects in color, flavor, and texture. ✓ Increases the moisture, fat, protein and thickness of cake	Increased	Increased	Increased	Arora et al. [173]

Grape pomace powder (20%)	Muffin	✓ Decreases the lightness of flour, while raises the lightness of crumb and crust of muffin. ✓ Enhance nutritional value.	Decreased	Increased	Increased	Baldan et al. [143]

Quince powder (10%)	Cake	✓ By adding higher amount reduces the volume, cohesiveness, resilience, and chewiness, ✓ Crumb shows the reduction in lightness and raises in redness/greenness value.	Increased	Increased	Increased	Salehi [174]

Apple pomace powder (15%) + wild sage seed gum	Cake	✓ Increases the nutritional value and shows good appearance.	Increased	—	Increased	Salehi [175]

Apple pomace powder (5%, 10%, 15%)	Cake	✓ By the addition of (WSSG) shows pseudoplastic and thixotropic behavior. ✓ Lower volume and greater density	Increased		Increased	Salehi and Aghajanzadeh [171]

Resistant maltodextrin > 20%	Cake and muffin	✓ Softer rice pancake. ✓ Decreases the cohesiveness, springiness, gumminess, and chewiness. ✓ The texture was not firm and friable	Increased	Decreased	Increased	Rakmai et al. [176]

Mosambi peel powder (6%)	Sausages and patties	✓ Improves storage stability ✓ Increases cohesiveness, tenderness, and strength of product. ✓ Decreases springiness	Increased	Increased	Decreased	Younis et al. [177]

Seaweed dietary fiber	Frankfurters	✓ Increases the cross‐linking density of the meat protein matrix and improves texture properties. ✓ Decreases springiness, resilience, cohesiveness, gumminess, and chewiness. ✓ Increases electrostatic interaction between MPs and potentially enhances the stability of intermolecular binding for the formation of denser and more compact three‐dimensional gel network structure.	—	Decreased	Increased	Yuan et al. [172]

wheat fiber mixture	Chicken sausages	✓ Increases moisture and lowers fat ✓ Decreases cooking yield, which indicates quality deterioration	Increased	Increased	—	Choe and Kim [165]

Foxtail millet	Paniyaram‐	✓ Increases the volume of batter and produces desirable soft, spongy texture to the product ✓ Increases the hardness when there is a complete substitution of millet into batter.	—	Increased	Increased	Khan and Peram [95]

Sour cherry pomace	Cake	✓ Minimized the negative effect in fat reduction. ✓ Extensibility, energy, weight loss, and cohesiveness values are found higher.	Increased	Decreased	Increased	Baskaya‐Sezer [178]

Cereals: amaranth, finger millet, pearl millet, sorghum	Idly	✓ Increases microbial activity and enhances enzyme activities in batter ✓ Improves the physicochemical and biochemical parameters.	—	Decreased	Increased	Rani et al. [70]

DF sources used in batter impact the texture, primarily through the egg yolk fraction, which significantly influences the structural properties [182]. The partial replacement of fat in bakery products adversely affects the textural and sensory properties of finished products [113]. Similarly, the texture of the chicken batter is influenced by factors such as the pre‐emulsification of sesame oil, fat content, and the amount of DF added. These factors affect parameters such as peak force, energy absorption, and elasticity [183]. Furthermore, the internal and external surfaces of baked products are influenced by lightness value and extrusion temperature, contributing to their overall appearance [84]. Textural parameters include hardness, cohesiveness, chewiness, and springiness. These parameters indicate the sample’s ability to regain its original shape after deformation. Gumminess and chewiness are determined by multiplying hardness, springiness, and cohesiveness, reflecting the effort required to chew the food to a consistency that is easy to swallow [184]. TPA determines cohesion by comparing positive force values under the first and second compressions [185]. Springiness and hardness indicate a food structure’s internal resistance to compression, reflecting the bonding strength within a three‐dimensional protein network [186]. Fermentation of gluten‐free cereals containing protein, lipids, and minerals with LABs enhances bioactive compounds and improves texture, shelf life, preservation, and sensory qualities in baked products [187]. The inclusion of hydrocolloids, such as XG, in batter products enhances shelf life without altering texture parameters during storage. Likewise, adding GG positively affects batter consistency and hardness, improving the cake and cookies’ texture with good sensory attributes [174]. Increasing the concentration of sodium bicarbonate and sodium tripolyphosphate in chicken meat batters, the textural properties (hardness, chewiness, springiness, and adhesiveness) increase with a slight effect on the protein and color of the sample [188].

Adding black glutinous rice flour (fiber) into the cake batter increases gumminess, chewiness, and firmness compared to a 100% wheat flour sample [189]. Extruded red rice flour (protein) in batter formulations affects the cake batter, and increasing barrel temperature produces a softer product texture, mainly due to greater starch breakdown [190]. In cake production, soya milk is used in place of eggs, which affects the batter texture by increasing firmness and viscosity and also affects the final product by increasing hardness, cohesiveness, springiness, and chewiness [191]. Aquafaba from chickpea canning (amino acids), when used as an egg replacer (ER) in cake, reduces the hardness of cake crumbs compared to control cakes prepared with egg white [192]. However, another study indicated that chickpeas as an egg substitute negatively affected the firmness of cake crumbs [193]. Likewise, using banana pomace (DF) as a replacer resulted in good textural attributes while decreasing the fat content in cake products [185]. Supplementation with chia seeds as ERs reduces resilience, indicating that the cake crumb takes longer to regain its shape after pressing [194]. Likewise, there were no significant differences in textural characteristics between the control and eggless cake batter made with banana and yoghurt [195]. The result of higher springiness values in fermented batter indicates its fresh, airy, and elasticized end products. Adding water‐soluble polysaccharides or hydrocolloids (HPMC) as an ER increases the cake quality with enhanced crumb color, texture, and mouthfeel.

The angel food cake made with egg white and fermented for 0, 3, 6, and 9 h shows that the cake has lower volume and less resilient texture. Also, it significantly enhances the firmness, stickiness, and tenderness of cake crumbs [145]. Incorporation of ∝‐amylase into honeycomb cake resulted in a gradual decrease in hardness and chewiness values, as ∝‐amylase possesses endohydrolytic activity, mainly breaking down the internal bonds of starch polymers, significantly decreasing their molecular size. Conversely, adding wheatgrass powder increased the hardness and chewiness of the cake sample [148]. Also, Aydogdu et al. [196] found that adding 5% oat and pea fiber in cake batter exhibits comparable volume density and texture compared to the control sample.

Conducting essential rheological tests in both linear and nonlinear domains can provide a detailed description of the viscoelastic properties of semiliquid networks [197]. The viscoelastic properties of macromolecules in batter mix, influenced by the addition of DF and water content, are determined by the particle size of flour and its density [198]. The addition of ADF and hydrocolloids improved the batter’s rheological properties, making its formulation more gel‐like. Also, Ref. [199] concluded that the fermentation in batter improves the protein, fiber, and rheological properties. The rheological behavior of batter can be improved within a small range of bacterial nanocellulose for bakery products [200]. Barley batter was significantly influenced by particle properties and water addition ratio [201]. Lin et al. [202] found that the effect of damaged starch can also influence the rheological properties of batter.

### 7.1. Effect of Viscosity on the Batter’s Formulated Product

Determining batter viscosity is crucial as it impacts specific gravity, bubble distribution, and the final product’s quality, particularly in volume and texture, by affecting air bubble expansion and stability during baking and frying. According to Huang and Yang [16], incorporating 20% Eucheuma powder into the cake batter enhanced the batter’s viscosity and viscoelasticity by affecting the texture and crumb color of the final product, whereas Salehi et al. [203] emphasize that sponge cake with 15% mushroom powder increases the batter viscosity from 12.55 to 18.20 Pa.s., and apparent viscosity decreases with a 10% addition. Salehi [175] concluded that using a higher level of apple powder in cake batter increases batter viscosity and the fiber content. Gholamian et al. [204] found that adding quince powder level (0%–20%) in cake batter increases the viscosity from 11.47 to 33.63 Pa.s, whereas Salehi and Aghajanzadeh [171] demonstrated that increasing the amount of strawberry powder in cake formulations increases specific gravity and dough viscosity. Using 30% carrot powder in cake batter increases the apparent viscosity and varies from 12.3 to 37.2 Pa.s [108].

By the concept of breadmaking, Tridtitanakiat et al. [205] revealed that adding soy okara flour to bread changes the rheological property of dough viscosity by affecting the dough’s consistency and increasing the hardness of the gluten‐free crumb. Also, breads made with 70% fermented cassava, 20% sweet potato, and 5% sorghum achieved optimal batter viscosity, resulting in higher volume and improved textural properties [167]. Yazar and Demirkesen [197] revealed that incorporating 15% or 30% sorghum flour into wheat tortillas increased viscosity and enhanced stress resistance in uniaxial tests. Ren et al. [206] demonstrate that the usage of double emulsion as a replacement for fat in meat products changes the texture properties, like a decrease in gum viscosity, chewiness, and hardness.

Seven different flours were added with seven batters for the preparation of pancake, which resulted in batter containing oats, legumes, rice, and corn showing greater weight loss, whereas batter prepared with wheat possessed less weight loss due to their higher protein and fiber contents (Lorento‐Bailo et al., 2021). The substitution of higher‐level pea proteins into cake batter by Bustillos et al. [77] resulted in the formation of a noncovalent network, which will be responsible for rheological behavior and final spongy cake structure. The pakoda batter depends upon the water added in the preparation of the batter and possesses shear‐thinning behavior (Kumar et al., 2019).

**FIGURE 6:**
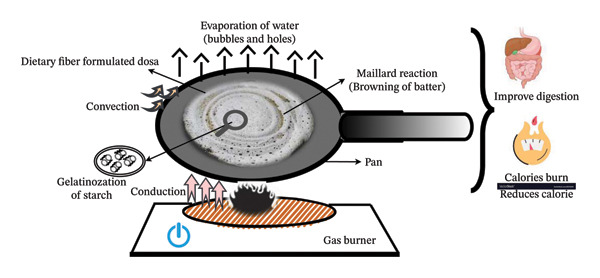
Thermal transformation of batter into dosa: Heat application triggers the biopolymers present in batter, resulting in textural changes, crisping, and formation of ambient patterns on the surface.

Adding egg white solids enhances the rheological properties of the batter, as well as the initial quality and storage stability of gluten‐free bread, with positive impacts on the crumb structure of bread [96]. Using 5% okara flour in gluten‐free roll batter resulted in lower deformation and flowability. At 10% addition, batter makes greater crumb firmness and reduces specific volume [205]. Inclusion of quince powder into cake batter found that the apparent viscosity decreases as the shear rate increases (pseudoplastic or shear‐thinning behavior), especially when the 20% formulation increases the viscosity of the cake batter [204]. The incorporation of 7.5 g foxtail millet into paniyaram batter resulted in better sensory characteristics than the 10‐g addition. Thus, the fermentation process improves the softness of the batter in the paniyaram recipe [207]. Likewise, using barnyard millet in dosa batter gives a good sensory score and high nutritional values. When millet flour increases, the thickness of the dosa decreases while frying starches get gelatinized and leading to a Maillard reaction as outlined in Figure 6, where the diameter was increased, resulting in an increased spread ratio [208].

Bhuiyan and Ngadi [20] studied that dipping chicken nuggets into the batter using different vegetable oils as a function of temperature (35°C−180°C) shows the viscosities of canola oil (42.49–4.65 MPa s) and soybean oil (38.63–3.31 MPa s), which enhance crispiness. Cappelli and Cini [209] studied the absence of gluten in cake batters, which resulted in lower gas holding capacity, and viscosity was closer to all samples. Changes in viscosity during cake making were linked to the transformation from foam to sponge form and to reduced shrinkage during baking, thereby improving the cake structure and quality. Also, using heat‐treated flour increases stiffness, viscosity, and resistance. A study by Yazici and Ozer [185] demonstrated that cake batter formulated with chia as an ER exhibited higher textural attribute values, including firmness, cohesion, uniformity, and thickness, when compared to batters made with banana or soy milk powder as ERs. Also, the eggless batter prepared for the cake shows higher viscosity values than the egg‐containing control batter. The addition of SPI or pea protein isolate (PPI), along with XG, which is rich in biological macromolecules, such as protein and peptides, further increased viscosity, while emulsifiers like monodiglycerides (MDGs) or sodium stearoyl lactylate (SL) reduced viscosity [210].

### 7.2. Effect of Antioxidant DF on the Batter’s Formulated Product

The concept of ADF combines the benefits of DF and antioxidants like polyphenols and carotenoids, where ADF containing over 50% fiber possesses DPPH free radical scavenging capacity equivalent to at least 50 mg of vitamin E per gram [211]. The linkage between phenolic compounds and DF through ionic, covalent, or hydrogen interactions is crucial for identifying potential antioxidant DFs. These interactions regulate the release and absorption of bioactive compounds in the gastrointestinal tract, influenced by the structure and functional groups of the antioxidants. To date, more than 8000 phenolic compounds have been described [212]. ADF can be metabolized by gut microbiota to enhance human health, inhibiting lipid oxidation, colorectal cancer, and intestinal tumorigenesis in mice models and preventing CVDs. It is also incorporated into foods to extend shelf life, prevent bread spoilage, and reduce lipid oxidation in fish and meat products [213].

ADF obtained from agro‐industrial by‐products and waste can be utilized at relatively low cost and in large quantities. DF obtained from the by‐products of fruits and vegetables (pomace, peels, and seeds) possesses greater physicochemical properties and soluble DF (33%) [214]. Likewise, the traditional by‐products of cereals such as wheat, corn, sorghum, and other legumes have been utilized as sources of fiber for the enrichment of food products. Bound polyphenols are essential for DF’s antioxidant and prebiotic properties [215].

According to Zhai et al. [216], fish nuggets prepared using wheat starch as an antioxidant DF source resulted in the frying of BBFNs at higher temperatures (160°C–180°C) for (60–120 s), increased water retention, and strengthened the gel layer formation by reducing oil penetration. Extreme frying conditions (190°C for 150 s) caused the structural breakdown of wheat gluten starch, leading to a rough, porous crust with higher oil absorption. RM is an SDF that improves the texture and softness of rice pancakes by lowering their GI. Also, the incorporation of jasmine flour by RM and sucrose by sucralose into pancakes makes it a low‐calorie diet for consumers [176]. The results revealed by Younis et al. [177] demonstrated that adding 6% mosambi peel powder to sausage and patties enhanced the fiber content and emulsion stability, improving hardness, firmness, and toughness but reducing springiness. However, Jeddou et al. [217] discovered that supplementing 10% potato pomace powder into a cake improves the quality by decreasing hardness, and the crumb color becomes darker with a saturated brown–orange color.

Frying of zucchini (fiber) with maltodextrin and ethanol batter results in a decrease in fat (12.8%) and an increase in the moisture content (27.3%) [218]. Likewise, incorporating protein and starch into the batter for dipping fish strips improves the textural properties and decreases the fat content [219]. Likewise, fat was reduced with better texture properties while incorporating fermented bamboo‐shoot DF into fish balls, as found by Zeng et al. [74]. Chicken nuggets’ fat content can be decreased by up to 33.2%, and there is no change in texture compared to the control, with the addition of quince seed gum and carvacrol microcapsule into the batter [50]. Also, Cengiz and Dogan [220] found that adding DRB to the batter decreased the fat content (4.9%) and crispiness of the chicken nuggets.

Adding dragon fruit peel to the batter for frying chicken nuggets, as reported by Madane et al. [221], improved the DF content, cooking yield, and odor score with an increase in lipid and color stability. It was reported that the inclusion of apple, orange, and carrot pomace in cake batter showed shear thinning and soft gel behavior with increased batter‐specific gravity and crumb hardness [156]. In a study by Kumari et al. [170], incorporating 10% buckwheat into parboiled rice has resulted in a higher rise in batter volume with increased total phenolic and flavonoid contents, which produces softer, fluffier idlis with a superior nutritional profile. The addition of up to 10% *Eucheuma* powder into cake increases the viscosity and viscoelastic of cake batters, and the sensory characteristics were satisfactory [16]. In a study by Huang et al. [169], the sample also affected the cake’s flow behavior and decreased the viscoelastic moduli after digestion, indicating a solid‐like behavior throughout the product. Replacement of wheat flour with 20% mushroom powder decreases batter density and specific volume, with an overall acceptability score of 7.35 [173].

## 8. Challenges and Future Work

Adding DF to batter‐based products poses several challenges, primarily related to texture, sensory characteristics, and process performance. On a textural basis, DF, particularly insoluble DF, tends to generate denser and harder crumbs and can decrease the overall volume of baked foods. The addition of carrot fiber to low‐fat cakes resulted in reduced batter viscosity, which had a negative impact on the volume and texture of the cake. The addition of buckwheat, beetroot, and flax fiber powders changed the rheological characteristics of rye–wheat dough, affecting its workability and the quality of the final product. In gluten‐free doughs, DF can disrupt the structural network, lowering dough elasticity. Yet some fibers, like psyllium husk and hydroxypropyl methylcellulose, have been found to improve crumb structure, providing potential solutions for gluten‐free baking.

Sensory acceptability is another significant challenge, with high levels of DF leading to the introduction of unwanted attributes such as bitterness, grittiness, or overdryness. Studies on rice bran and sugarcane bagasse addition to bread products showed that such fibers have the potential to compromise flavor and mouthfeel. Industrial processing issues also arise, leading to the high water‐holding capacity of DF, which can compromise dough hydration and mixing tolerance, thereby affecting starch gelatinization and retrogradation. These cause inferior texture and shortening of shelf life in baked goods with added fiber. The choice of fibers and the processing methods plays a key role in overcoming these challenges, as excessive fiber can lead to undesirable mouthfeel or degradability. Furthermore, ensuring the stability of functional and bioactive components of DF during high‐temperature processing adds another layer of complexity. To overcome these challenges, future studies should focus on optimizing the blend of various fiber types, for example, combining soluble and insoluble fibers, to find a balance between nutritional and technological performance. Future research should focus on the optimization of rheological properties of DF added to baked goods, with the aim of maximizing health benefits. Innovation in fermentation, the enzymatic modification of DF will pave the way for compatibility in batter‐based products. Also, developing interactions within the DF matrix will aid in the formulation of more consistent and acceptable products. Additionally, advances in processing interventions, such as high‐pressure processing, ultrasound‐assisted mixing, and extrusion techniques, can improve texture and overall consumer acceptability. Multidisciplinary collaboration between food technologists, nutritionists, and sensory scientists will be crucial to address the dual goals of health and consumer satisfaction. Lastly, consumer education and market strategies promoting the health benefits of natural DF‐enriched batter‐based products will be vital for wider acceptance and commercialization.

## 9. Conclusion

Healthier batter formulation by natural DF through optimizing rheological properties has a warm reception by consumers if the batter and coatings supplemented with biopolymers such as protein isolate, starch, pectin, cellulose, lignin, inulin, hemicellulose (arabinoxylans), and DF can significantly enhance the batter viscosity and stability, resulting in better texture and organoleptic characteristics, along with improved nutritional content. Many researchers have focused on the rheological properties of batter systems incorporated with different flours. The characteristics of fermented batter enriched with dietary components have been discussed in this work. The use of different LAB strains during fermentation strengthens biological macromolecules, and the batter’s functional properties pave the way for desirable texture and good overall sensory attributes.

Supplementation with different levels of natural DF sources in batter formulations influences the biopolymers, including protein, carbohydrate, starch, and fiber. It also influences oil uptake, batter volume, batter density, and batter stability during fermentation and during baking and frying. The advancement in the fermentation process will lead to better optimization in the rheological properties of batter, enabling the formulation of healthier and more sustainable food products. It also found that fermentation period and rheological properties are interrelated in batter formulations. However, the interaction of LAB strains with DF‐enriched components in enhancing the nutritional and textural properties of batter‐based products is still unclear. The gap in the literature includes the unrevealing of the rheological behavior of batter formulated with fermented DF. Further study should be conducted on the fermentation of dietary components and optimization of their rheological properties for healthier diet management. Specifically, the influence of fermentation on the micropattern, composition, and interactions between the batter and food products warrants further investigation. Future research should focus on the interplay between fermentation duration and rheological properties in batters with diverse DF sources, developing methods to optimize the fermentation process to enhance batter stability and functionality under various cooking conditions. Addressing these gaps will pave the way for healthier, more sustainable food products and advance dietary management strategies through optimized batter systems.

## Author Contributions

Sangeeth Raj A.: writing–original draft and writing–review and editing. Praveen Kumar Dubey: conceptualization, administration, methodology, writing–original draft, data curation, supervision, validation, visualization, and writing–review and editing. Hridyesh Pandey: validation, visualization, and data curation. Ashutosh Upadhyay: data curation, validation, visualization, and supervision.

## Funding

This research did not receive any specific grant from funding agencies in the public, commercial, or not‐for‐profit sectors.

## Conflicts of Interest

The authors declare no conflicts of interest.

## Data Availability

The data that support the findings of this study are available from the corresponding author upon reasonable request.
